# Unveiling age-differentiated pathways: spiritual well-being links to quality of life in breast cancer survivors through network analysis

**DOI:** 10.3389/fpubh.2026.1782688

**Published:** 2026-06-12

**Authors:** Cheng Yang, Qin Zeng, Jiawei Li

**Affiliations:** 1 Department of Pediatric Intensive Care Unit Nursing, West China Second University Hospital, Sichuan University, Chengdu, China; 2 Department of Pediatric Intensive Care Unit Nursing, WCSUH-Tianfu·Sichuan Provincial Children's Hospital, Meishan, China; 3 Key Laboratory of Birth Defects and Related Diseases of Women and Children, Sichuan University, Ministry of Education, Chengdu, China; 4 Department of Gastroenterology Nursing, West China Second University Hospital, Sichuan University, Chengdu, China; 5 Department of Pediatric Outpatient Nursing, West China Second University Hospital, Sichuan University, Chengdu, China

**Keywords:** breast cancer survivors, bridge symptoms, network analysis, quality of life, spiritual well-being, survivorship care

## Abstract

**Background:**

Enhancing the quality of life (QoL) of breast cancer survivors is a key public health priority. Spiritual well-being (SpWB) is recognized as a correlate, but its systemic role in connecting different life domains and how this varies by age remain unclear, hindering the generation of hypotheses for tailored support programs.

**Methods:**

We conducted a cross-sectional study of 302 breast cancer patients (stages I-III) in active treatment. Using psychometric network analysis, we mapped the interconnections between QoL (FACT-B scale) and SpWB (FACIT-Sp-12). Centrality and bridge metrics identified key nodes, and comparisons were made between younger (≤50) and older (>50) patients.

**Results:**

SpWB was the strongest connector among physical, emotional, social, and functional QoL domains. The most central concerns were fear of disease progression and difficulty accepting the disease. Age-based differences appeared in network connectivity strength. Younger patients showed tighter links among identity, emotional distress, and other domains, suggesting greater vulnerability to interacting difficulties. Older patients relied more on separate social support nodes.

**Conclusion:**

This study provides a systemic, person-centered map of resilience in a cohort of Chinese breast cancer patients. SpWB is not merely a personal resource but a central hub associated with broad aspects of well-being in this sample. The marked age differences call for age-stratified support strategies: younger survivors may benefit from integrated psycho-spiritual interventions targeting identity and fear, while older survivors may profit from strengthened social support networks. These preliminary findings suggest that integrating spiritual health assessment into survivorship care may be worth considering, pending validation in diverse populations.

## 1 Introduction

Breast cancer survivorship is a growing public health concern. The disease affects many aspects of women’s lives and reduces quality of life (QoL) (1, 2). Better post-treatment QoL is linked to improved clinical outcomes and resilience, but healthcare often overlooks this (1). Spiritual well-being (SpWB) is associated with QoL. For example, meaning (OR = 0.436) and peace (OR = 0.303) show positive links to life satisfaction (3–5). This relationship warrants particular attention since unaddressed spiritual needs may compromise well-being, whereas their proper management can optimize overall QoL (6).

In addition to spiritual well-being, fear of cancer recurrence (FCR) represents a pervasive psychological challenge among breast cancer survivors, with prevalence rates reaching up to 73% (7). A recent systematic review synthesized qualitative evidence on coping strategies for FCR, identifying four major themes: seeking support, transforming health behaviors and lifestyle, avoidance and emotional detachment, and building resilience and emotional strength (7). These findings underscore the multifaceted nature of survivors’ responses to FCR and highlight the importance of tailored support that addresses individual coping styles. Understanding how FCR interrelates with other QoL domains, such as emotional and functional well-being, is essential for designing comprehensive interventions.

Distress symptoms and psychological factors in breast cancer interact in complex ways that are difficult to study with conventional methods. Network analysis (NA) provides a useful framework. It uses graph theory to (a) show symptom patterns and (b) identify central or bridge nodes that could be intervention targets (8–10). This approach enables examination of interconnected systems involving anxiety, depression, and QoL components, revealing critical bridge symptoms (e.g., family functioning, social support) that mediate psychological-outcome relationships (11–13). By providing symptom-level resolution of cancer complexity, NA overcomes traditional methodological constraints and facilitates personalized clinical strategy development (9, 14).

Epidemiological evidence underscores the global imperative for such innovations. In emerging economies, breast cancer management requires urgent assessment of prognostic factors, healthcare quality, and resource allocation equity to improve survival rates (15). Worldwide epidemiological patterns reveal that while advanced breast cancer patients now experience prolonged survival, they concurrently endure exacerbated spiritual distress and diminished QoL without appropriate interventions (16). These findings necessitate urgent investigation of spiritual health networks, which may reveal novel pathways for QoL enhancement and care disparity mitigation.

The impact of breast cancer extends beyond the individual patient, profoundly affecting partners’ sexual and emotional well-being. Maleki et al. (17) found that husbands of breast cancer survivors often experience unfulfilled sexual expectations, diminished sexual desire, and a lack of supportive communication, which can strain marital relationships and compound the survivor’ s distress. Furthermore, survivors themselves, particularly those of reproductive age, report significant sexual dysfunction, including decreased libido, vaginal dryness, and body image disturbances, often exacerbated by insufficient information and support from healthcare providers (18). These findings highlight the critical need to address sexual health and couple dynamics as integral components of comprehensive survivorship care.

While the association between SpWB and QoL is established, understanding its functional architecture and actionable mechanisms during the vulnerable period of active treatment is crucial for designing effective early interventions and public health support strategies. Conventional statistical methods, which treat these constructs as monolithic entities, are ill-suited to uncover the complex, non-linear interactions at the symptom level that may underlie resilience and distress. This study used psychometric network analysis for three aims: ① To model how specific QoL and SpWB components interconnect; ② To identify the most central and bridging nodes in this network (candidate targets that show strong associations and could be prioritized in future research, though causal inference requires longitudinal designs); ③ To test if this network differs by age.

## 2 Research methods

### 2.1 Study design

This study employed a cross-sectional design.

### 2.2 Study participants

#### 2.2.1 Sampling method

The study consecutively enrolled breast cancer patients who met the inclusion criteria at a tertiary Grade A hospital in Sichuan Province between May and December 2023. To enhance sample representativeness and account for the impact of chemotherapy cycles on quality of life (19, 20), data were collected at multiple chemotherapy time points. Specifically: ① For patients receiving 4 chemotherapy cycles, one session was randomly selected from cycles 1–4; ② For 6-cycle regimens, one session was selected from cycles 5–6; ③ For 8-cycle regimens, one session was selected from cycles 7–8; ④ For patients with chemotherapy cycles other than 4, 6, or 8, data were collected during one of the final 1–2 cycles. This pragmatic approach was designed to capture the variance in QoL during active treatment by sampling across different phases of chemotherapy.

#### 2.2.2 Diagnostic and inclusion/exclusion criteria

Diagnostic criteria: patients were included based on pathological diagnosis of stage I, II, or III breast cancer according to the 8th edition of the American Joint Committee on Cancer (AJCC) TNM staging system (21).

##### 2.2.2.1 Inclusion criteria

(1) Female patients pathologically diagnosed with breast cancer;
(2) Aged ≥18 years;
(3) Completed surgery and at least one chemotherapy cycle;
(4) Voluntarily participated in the study;
(5) Cognitively intact with normal verbal ability.

##### 2.2.2.2 Exclusion criteria

(1) Patients with cancer recurrence;
(2) History or current diagnosis of other malignancies;
(3) Comorbid major systemic diseases.

##### 2.2.2.3 Elimination criteria

(1) Questionnaires with >20% missing data were excluded;
(2) Self-contradictory responses were excluded.

Questionnaires with ≤20% missing items were retained for analysis, with missing values handled as described in Section 2.5 (Missing data handling).

#### 2.2.3 Sample size estimation

The sample size was calculated using the formula for cross-sectional studies: *N* = (*Ua* × *σ*/*δ*)^2^, where *Ua* is 1.96 (two-tailed *α* = 0.05), *σ* represents the standard deviation, and *δ* denotes the permissible absolute error (error of mean or proportion). Since σ is typically unknown, it is commonly estimated using sample statistics such as the sample mean (or proportion *p*) and sample standard deviation *S*, which are often derived from pilot studies, literature reviews, or empirical experience (22). In the preliminary survey of this study, the *S* value (standard deviation of the total score on the quality-of-life scale) was determined to be 14, based on prior literature. The *δ* value was set at 2. Substituting these values into the formula yields: *N* = (1.96 × 14/2)^2^ = 188 participants. Accounting for a 20% dropout rate, the final required sample size was adjusted to 226 participants. Notably, subsequent network stability analyses confirmed the adequacy of this sample size for network estimation, with correlation stability coefficients exceeding recommended thresholds.

#### 2.2.4 Age group classification

The age cutoff of 50 years was selected based on two considerations. First, 50 years approximates the mean age of natural menopause in Chinese women, and breast cancer burden varies significantly across premenopausal, perimenopausal, and postmenopausal stages (23). Second, this cutoff is commonly used in breast cancer survivorship research to distinguish younger from older patients, facilitating cross-study comparisons (24).

### 2.3 Research instruments

#### 2.3.1 General information questionnaire

Designed by the researchers, this questionnaire includes 17 items covering general demographic data (e.g., age, education level, marital status) and disease/treatment-related information (e.g., disease duration, tumor stage, surgical approach).

#### 2.3.2 Functional Assessment of Cancer Therapy-Breast (FACT-B)

The Functional Assessment of Cancer Therapy-Breast (FACT-B) scale was developed by Cella (25) in the United States and later translated into Chinese by Wang et al. (26), with a reported overall Cronbach’s alpha coefficient of 0.89. This scale comprises 36 items across five dimensions: physical well-being, social/family well-being, emotional well-being, functional well-being, and additional concerns. Each item is rated on a 5-point Likert scale (0–5), ranging from “not at all” to “very much,” with a total score ranging from 0 to 144. Higher scores indicate better quality of life. In the pilot study, the Cronbach’s alpha coefficient was 0.85, while in the formal survey, it was 0.94.

#### 2.3.3 Chinese version of the Functional Assessment of Chronic Illness Therapy-Spiritual Well-Being Scale (FACIT-Sp-12)

The Chinese version of the Functional Assessment of Chronic Illness Therapy-Spiritual Well-Being Scale (FACIT-Sp-12) was translated and validated by Liu et al. (27), with a content validity index of 0.90 and a Cronbach’s alpha coefficient of 0.83. This 12-item self-report scale assesses three dimensions: peace, meaning, and faith, using a 5-point Likert scale (0–4). Spiritual well-being is categorized as low (<24 points), moderate (24–35 points), or high (>36 points). In the pilot study, the Cronbach’s alpha coefficient was 0.887, while in the formal survey, it was 0.90.

### 2.4 Data collection and quality control

#### 2.4.1 Data collection

During the pilot survey, 25 eligible patients tested the questionnaire. Ambiguous items were revised, e.g., modifying “supervisor” to “supervisor/elder” in one item and “sex life” to “sex life/intimate relationships” in another. The formal survey used anonymous paper questionnaires (*n* = 303) administered by trained professionals in a Chinese oncology ward, with nurse assistance when needed. Participants received study explanations and chose paper format only. Researchers clarified questions and checked for completeness upon collection.

#### 2.4.2 Quality control

A scientifically sound protocol was developed with expert consultations. Researchers coordinated with hospitals, used standardized questionnaires, and verified completeness. Electronic tools enabled real-time monitoring and on-site guidance. Appropriate statistical methods ensured reliable results. Quality control was maintained through study design, data collection, and analysis safeguards.

### 2.5 Statistical analysis

Data analysis in this study was conducted using R software (version 4.4.3), primarily utilizing the ‘qgraph’, ‘NetworkComparisonTest’, and ‘bootnet’ packages for network analysis. Descriptive statistics were first performed for all variables, with continuous variables presented as mean ± standard deviation. All demographic data were optimized into categorical variables and thus expressed as frequencies and percentages. Intergroup comparisons of demographic characteristics were assessed using the *χ*^2^ test, with results reported as frequencies and percentages; a *p* < 0.05 was considered statistically significant. To evaluate the discriminative validity of items in the FACT-B scale, standard deviations were calculated as measures of variability, and inter-item correlations were examined to test for redundancy, ensuring the independence of each scale item.

Missing data handling. For the 302 eligible questionnaires retained after applying the elimination criteria (Section 2.2.2), the proportion of missing items across all 37 nodes was <1%. For the small number of missing responses (≤20% of items per questionnaire), person-mean imputation was applied at the scale level. Specifically, for each of the six scales (Physical Well-Being, Social/Family Well-Being, Emotional Well-Being, Functional Well-Being, Additional Concerns, and Spiritual Well-Being), the mean of the available items completed by the same participant was calculated and used to replace the missing item(s) within that same scale. This approach follows standard recommendations for handling sporadic missing data in quality-of-life research (28).

#### 2.5.1 Data suitability assessment

Prior to network analysis, the suitability of the 37 items for network estimation was evaluated. Network analysis requires that variables exhibit some degree of correlation but not severe multicollinearity. The suitability of the data was assessed using the following criteria:

(1) Item discriminability: standard deviations of all items were examined to ensure adequate variability and the absence of low-information items (criteria: SD > 0.3).
(2) Multicollinearity assessment: inter-item zero-order correlations were inspected to rule out severe multicollinearity, with correlations >0.85 considered indicative of potential redundancy and unsuitable for network estimation (29).
(3) Sample size adequacy: the sample size was evaluated against the rule-of-thumb requirement for network analysis, which recommends 5–10 times the number of variables (29).
(4) Network stability: the robustness of the estimated network would be subsequently assessed using the correlation stability coefficient (CS-coefficient), with values >0.5 indicating stable centrality estimates.

#### 2.5.2 Network structure estimation

A Gaussian Graphical Model (GGM) was estimated using the qgraph package in R. LASSO regularization was applied to reduce noise and highlight core associations, with model sparsity optimized using the Extended Bayesian Information Criterion (EBIC). The EBIC hyperparameter *γ* was set to 0.5, following the default recommendation for psychometric network analysis to balance sparsity and sensitivity (30). The Fruchterman–Reingold algorithm was employed to position nodes, placing more central items at the core of the network. Nodes represented the 37 items from the FACT-B and FACIT-Sp-12 scales, and edges represented partial correlations (green for positive, red for negative), revealing the multidimensional structure of the data (29, 31). The Spiritual Well-Being dimension was operationalized using the total score of the FACIT-Sp-12 scale (range 0–48), which was entered as a single aggregate node. This approach was chosen to maintain model parsimony and to test the overarching hypothesis that global spiritual well-being serves as a cross-domain bridge within the overall quality-of-life network, rather than to distinguish among its subdimensions (meaning, peace, faith). Age-group differences in network structure were examined using the Network Comparison Test.

#### 2.5.3 Network characterization

Network properties were characterized by three metrics: Node Strength, Bridge Strength, and Node Predictability. Node Strength is the sum of absolute weights of all edges connected to a node, reflecting its overall influence in the network. Bridge Strength is the sum of edge weights connecting a node to other dimensional nodes, evaluating cross-dimensional interactions (32). Node Predictability quantifies how well a specific item can be predicted by other nodes in the network (33). These metrics collectively reveal the complex interaction mechanisms between quality of life and spiritual health in breast cancer patients.

#### 2.5.4 Network validation

To ensure reliability and robustness of network analysis results, multiple validation methods were employed for comprehensive network quality assessment (29). First, edge accuracy analysis examined network structure reliability by calculating 95% nonparametric confidence intervals (CIs) for edge weights through 1,000 bootstrap samples—narrower CIs indicate higher estimation precision. Second, centrality stability analysis evaluated metric stability using the correlation stability coefficient (CS coefficient) to quantify node strength reproducibility: results are considered acceptably stable when CS > 0.25, and ideally stable when >0.5. Finally, bootstrap difference tests compared statistical differences in edge weights and node strengths, with 95% CIs excluding zero considered significant (*p* < 0.05). These methods support the reliability of our network analysis results. The very low rate of missing data (<1%) combined with the person-mean imputation procedure ensured that missing values did not meaningfully affect the estimated network structure or centrality stability.

## 3 Results

### 3.1 Sample characteristics and demographic analysis

Table 1 summarizes baseline characteristics of 302 breast cancer patients: 60.9% ≤ 50 years, 39.1% > 50 years. Most were Han (94.7%), married (93.0%), urban residents (70.5%), with high family support (77.5%). Education levels varied (29.1% bachelor’s+). Disease characteristics: 91.4% duration <1 year; stages I (37.7%), II (47.7%), III (14.6%). Treatments: 87.1% surgery + chemotherapy (mastectomy 49.3%, breast-conserving 33.8%). Supplementary Table 1 shows age-group differences (≤50 vs. > 50) in education, employment, and surgical approach (*p* < 0.05).

**Table 1:** Characteristics of breast cancer patients (*N* = 302).

Characteristics	Frequency (*N*)	Percentage (%)
Age (years)		
≤50	184	60.9%
>50	118	39.1%
Ethnicity		
Han	286	94.7%
Others	16	5.3%
Marital status^a^		
Married	281	93.0%
Single	21	7.0%
Educational level		
Bachelor’s degree or above	88	29.1%
Junior college	52	17.2%
Middle school and below	162	53.6%
Religious beliefs^b^		
Yes	278	92.1%
No	24	7.9%
Current work status		
Retired	106	35.1%
Employed	105	34.8%
Unemployed	91	30.1%
Family residence location		
Urban	213	70.5%
Rural	51	16.9%
Town	38	12.6%
Residential status		
Living with family or friends	291	96.4%
Living alone	11	3.6%
Level of family care and support since the illness onset		
Very much	234	77.5%
Quite a lot	56	18.5%
Relatively little	12	4.0%
Personality types		
Ambivert type	134	44.4%
Extroversion tendency type	84	27.8%
Introversion tendency type	41	13.6%
Typical extrovert type	36	11.9%
Typical introvert type	7	2.3%
Per capita monthly household income		
5,000 yuan and below	117	38.7%
5,001–8,000 yuan	73	24.2%
8,001 yuan and above	112	37.1%
Disease duration (years)		
<1	276	91.4%
≥1	26	8.6%
Tumor stage		
Stage I	114	37.7%
Stage II	144	47.7%
Stage III	44	14.6%
Sequence of surgery and chemotherapy		
Primary debulking surgery (PDS) followed by chemotherapy	263	87.1%
Neoadjuvant chemotherapy (NAC) followed by surgery	39	12.9%
Breast surgery approaches		
Mastectomy	149	49.3%
Breast-conserving surgery	102	33.8%
Breast reconstruction	51	16.9%
The total number of chemotherapy cycles required to be received		
8	91	30.1%
6	83	27.5%
4	126	41.7%
Other	2	0.7%
Chemotherapy completion status		
Completed	95	31.5%
Uncompleted	207	68.5%

a Single indicated separated, divorced, widowed, or never married, and married indicated married or partnered.

b Religious affiliations included Buddhism (22 individuals) and Taoism (2 individuals).

### 3.2 Measurement tool analysis

For the FACT-B scale (Supplementary Table 2) and FACIT-Sp-12 scale (total score: 33.58 ± 7.92), all 37 items demonstrated standard deviations within reasonable ranges (no low-information items), and inter-item correlations were <0.25, confirming good discriminative validity and independence.

### 3.3 Network characteristics analysis

#### 3.3.1 Network structure estimation and centrality features

**Figure 1:**
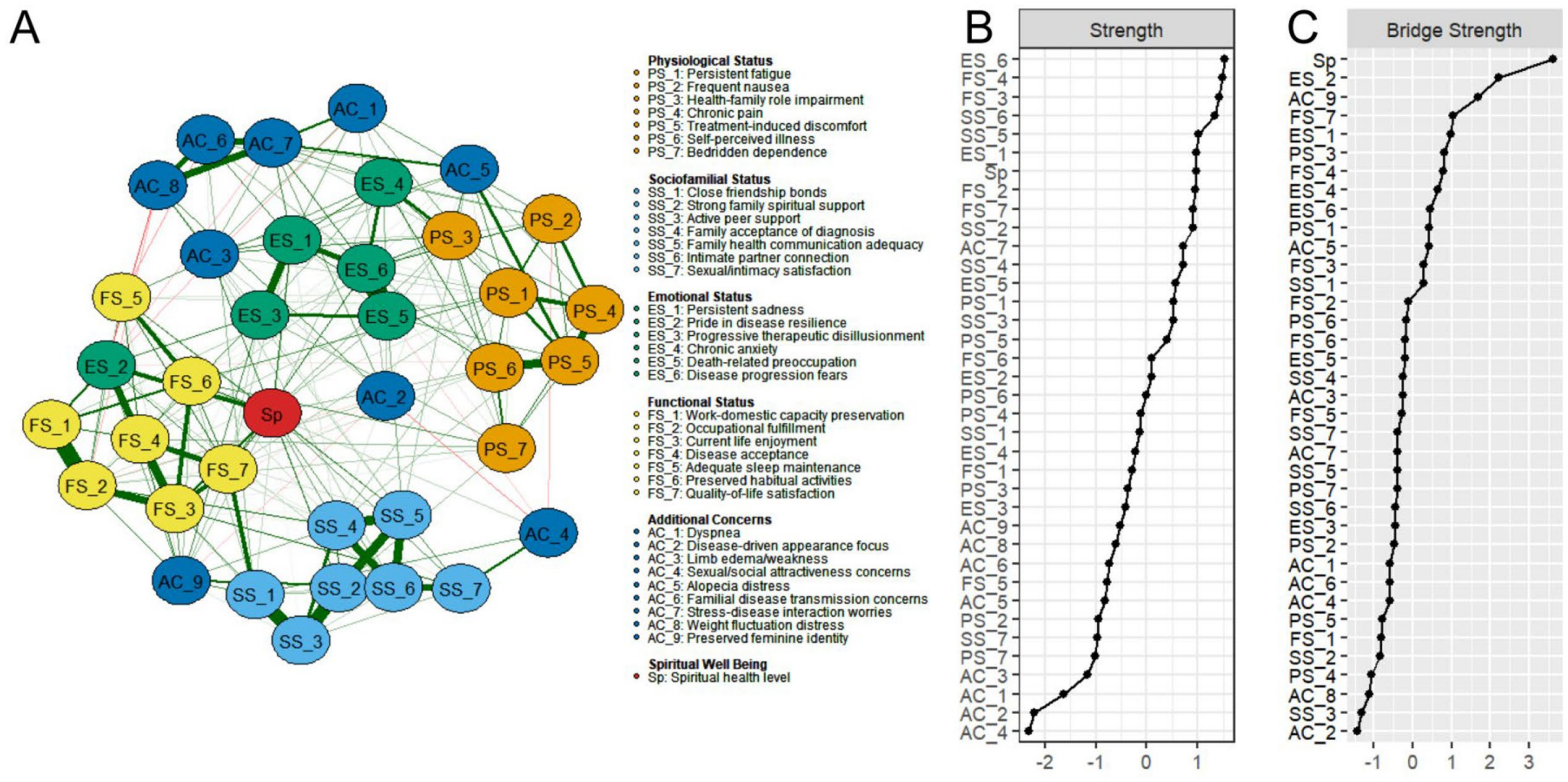
Network structure and centrality indices. **(A)** Network of 37 nodes. Green edges indicate positive partial correlations; edge thickness reflects correlation strength. **(B)** Strength centrality (sum of all edge weights connected to a node). Higher values = greater overall influence. ES_6 (fear of disease progression) is most central (strength = 1.522). **(C)** Bridge strength centrality (sum of edges connecting a node to nodes in other QoL domains). Higher values = stronger cross-domain connectivity. SP (spiritual well-being) shows the highest bridge strength (3.601), indicating it links physical, emotional, social, and functional domains.

Figure 1A shows the network. In this undirected graph, green edges indicate positive partial correlations, and edge thickness reflects correlation strength. The network has 37 nodes: 36 items from the FACT-B scale (physical, social/family, emotional, functional, and additional concerns) and one SP node (spiritual well-being). The network has 227 non-zero edges (34.1% of all possible edges). The average edge weight is 0.023. This sparsity does not mean the domains are independent. Instead, most connections go through a few hub nodes: SP, fear of disease progression (ES_6), and disease acceptance (FS_4). These hubs link different life domains. Because they are associated with multiple domains, they could be targets for future studies.

Strength centrality results (Figure 1B, Supplementary Table 3) reveal that ES_6 (“Disease progression fears”) from the Emotional Status dimension exhibits the highest influence (Strength = 1.522), followed by FS_4 (“Disease acceptance,” Strength = 1.492) and FS_3 (“Current life enjoyment,” Strength = 1.432) from the Functional Status dimension.

Bridge strength measures how strongly a node connects to nodes in other QoL domains (e.g., emotional to functional). Bridge strength analysis (Figure 1C, Supplementary Table 3) identifies SP (“Spiritual health level,” Strength = 3.601) from the Spiritual Well-Being dimension as the most pivotal bridging node. This is followed by ES_2 (“Pride in disease resilience,” Strength = 2.209) from the Emotional Status dimension and AC_9 (“Preserved feminine identity,” Strength = 1.691) from the Additional Concerns dimension.

**Figure 2:**
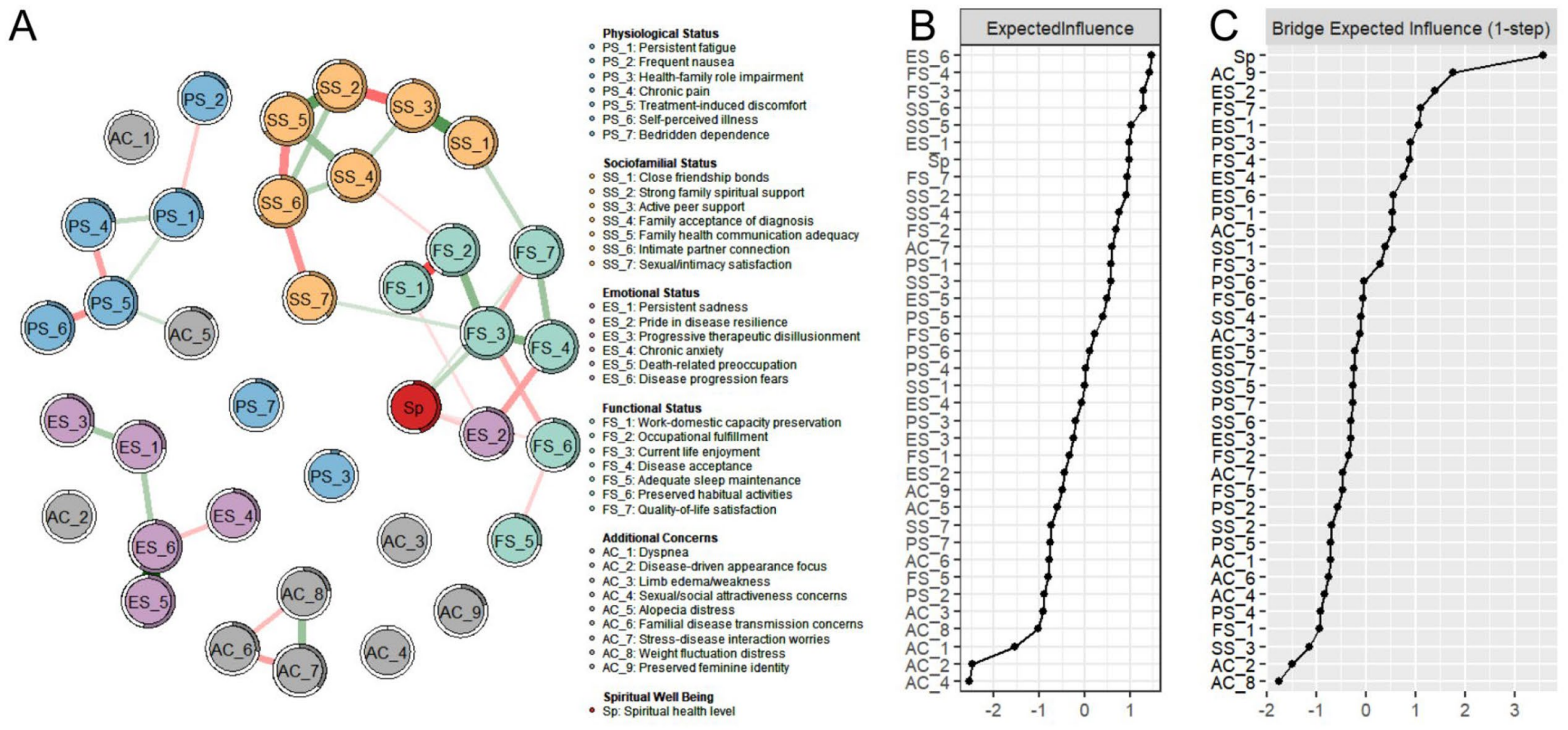
Node predictability and expected influence. **(A)** Same network as Figure 1A, with nodes colored by predictability (R^2^, the proportion of variance explained by other nodes). Darker blue = higher predictability (e.g., SS_2_, family spiritual support, *R*^2^ = 0.698). **(B)** Expected influence (sum of edge weights including negative edges; here identical to strength). **(C)** One-step bridge expected influence. SP again has the highest value (3.601), confirming its bridging role.

Predictability (*R*^2^) indicates how much of a node’s variance is explained by all other nodes in the network. In Figure 2A, darker blue nodes have higher *R*^2^. Predictability analysis (Figure 2, Supplementary Table 3) demonstrates that the predictability values for the 37 nodes range from 0 to 0.698. SS_2 (“Strong family spiritual support,” *R*^2^ = 0.698) from the Sociofamilial Status dimension is the most predictable node, with 69.8% of its variance explainable by other items in the network. This is followed by SS_5 (“Family health communication adequacy,” *R*^2^ = 0.667) and FS_3 (“Current life enjoyment,” *R*^2^ = 0.664) from the Functional Status dimension.

#### 3.3.2 Analysis of network characteristics differences across age groups

Network analysis using Gaussian graphical models showed no significant structural differences between age groups (≤50 vs. > 50) (Supplementary Table 4). The Network Invariance Test (*M* = 0.205, *p* = 0.831) (Supplementary Figure 1) and Global Strength Invariance Test (S = 4.050, *p* = 0.814) confirmed that the global connectivity pattern—i.e., which specific nodes are connected to each other—is statistically indistinguishable between younger and older survivors. Only one edge (SS_1-SS_5, *p* = 0.023) showed a significant difference among 37 node pairs. Centrality Invariance Test revealed no node-level differences in node strength (all *p* > 0.05). Taken together, these results indicate that the “skeleton” of the network is largely conserved across age groups.

However, a different picture emerged when examining bridge strength, which quantifies the intensity with which nodes connect across different life domains (e.g., emotional to functional). Bridge strength analysis revealed significantly higher overall bridge centrality in the ≤50 group compared to the >50 group (*t* = 9.30, *p* < 0.001, Cohen’s *d* = 1.656). This indicates that while the pattern of connections is similar, the strength of cross-domain connectivity is substantially greater in younger survivors—their well-being system is more “tightly interwoven.” Specifically, 34 out of 37 nodes showed higher bridge strength in the younger group (Supplementary Figure 2). Top differing nodes included AC_9 (*Δ* = 0.754) and ES_1 (*Δ* = 0.675). Only SS_6 showed higher centrality in >50 group (*Δ* = −0.048). Functional analysis revealed 22 nodes had zero centrality in >50 group, with largest differences in Emotional Status (6/7 nodes) and Additional Concerns (4/9 nodes) domains. Thus, the connection pattern is similar across ages, but the connection strength is greater in younger survivors.

### 3.4 Network accuracy and stability analysis

#### 3.4.1 Edge weight accuracy

This study validated breast cancer patients’ quality of life and spiritual health network analysis. Bootstrap analysis showed weak connections (median strength 0.0221, below 0.1 threshold), with narrow confidence intervals confirming significance. The sparse network suggests indirect node associations and modular substructures. Core connections were stable, while weaker edges had wider confidence intervals, indicating estimation uncertainty.

#### 3.4.2 Centrality stability

**Figure 3:**
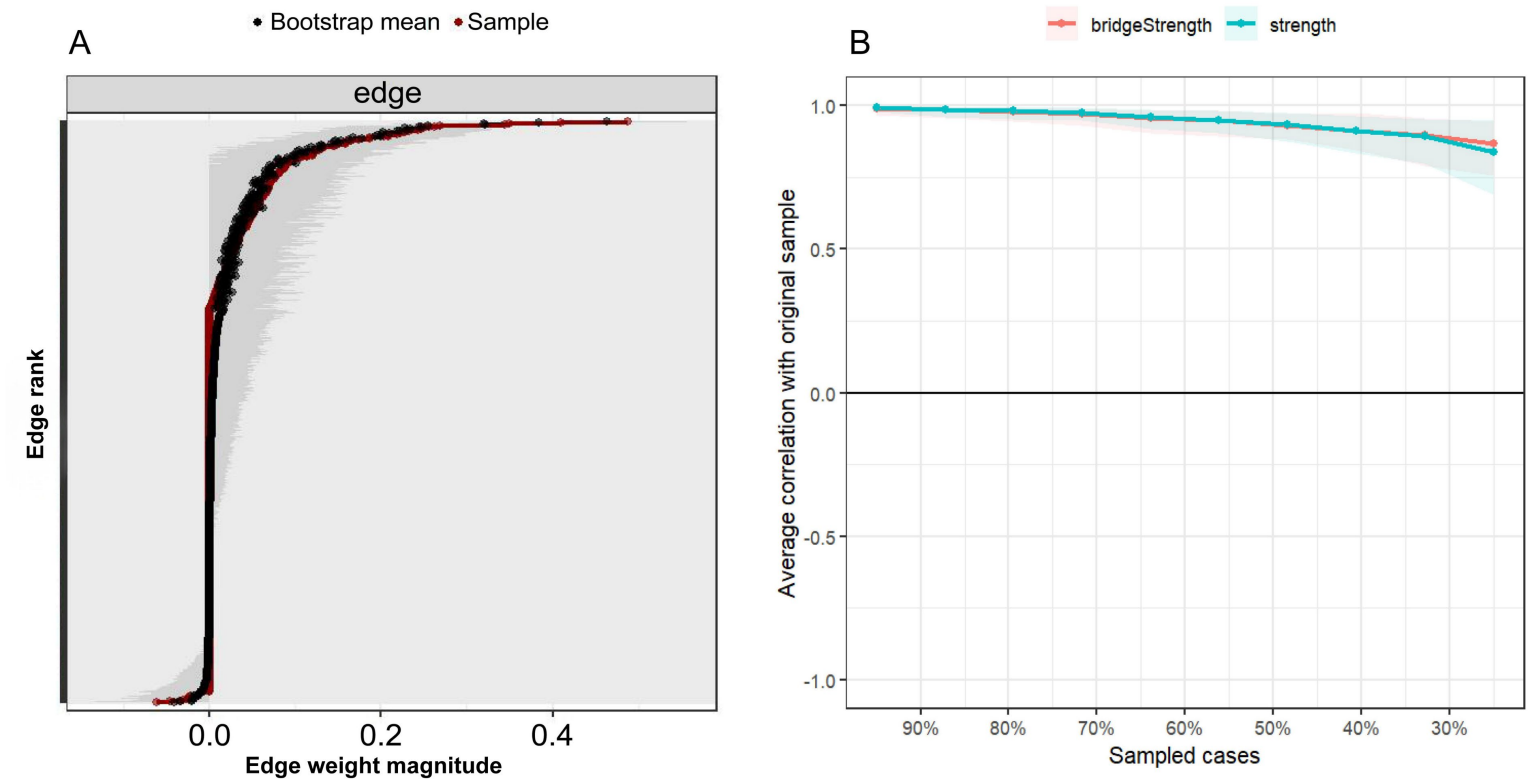
Edge accuracy and centrality stability. **(A)** Bootstrapped 95% confidence intervals (CIs) for edge weights. Narrow CIs for strong edges indicate precise estimation; wider CIs for weak edges reflect sampling uncertainty. **(B)** Correlation stability (CS) coefficients for strength centrality (left) and bridge strength (right). CS = 0.75 (well above the recommended threshold of 0.5) confirms that centrality rankings remain stable even when up to 75% of the sample is removed.

Stability analysis of centrality metrics (Figure 3B) demonstrated that the correlation stability coefficients (CS) for both strength centrality and bridge strength reached 0.75, significantly exceeding the recommended threshold of 0.5. This means that the ranking of node centralities would remain stable even if up to 75% of the sample were randomly removed, indicating excellent robustness. The result further solidifies the pivotal roles of key nodes [e.g., ES_6 (Disease progression fears, Strength = 1.522, Figure 1B) and SP (Spiritual health level, Bridge strength = 3.601, Figure 1C)] within the network.

### 3.5 Network characteristics difference testing

This study analyzed network characteristics in breast cancer patients, revealing structural differences between quality of life (QoL) and spiritual health networks. Edge weight analysis identified 20 significant core connections, with the strongest being: ① Death-related preoccupation—Disease progression fears (*M* = 0.486); ② Work capacity—Occupational fulfillment (*M* = 0.359); ③ Friendship bonds—Peer support (*M* = 0.337); ④ Family spiritual support—Health communication (*M* = 0.296); ⑤ Family support—Peer support (*M* = 0.289). All edge weights ranged 0.172–0.486. Node centrality testing showed Disease progression fears (Strength = 1.522), Disease acceptance (1.492), and Current life enjoyment (1.432) as most central. Bridge analysis revealed Spiritual health level (3.601), Pride in disease resilience (2.209), and Preserved feminine identity (1.691) as key cross-dimensional connectors. These findings highlight the interconnected roles of emotional, functional and spiritual factors in patients’ QoL and spiritual health networks (Supplementary Figures 3–5).

## 4 Discussion

Improving QoL for breast cancer survivors is important. Although SpWB is linked to QoL, its role in the overall well-being system is not well understood. This study used network analysis to map how QoL and SpWB are interconnected. In this cohort, we identified candidate targets and age differences. These findings may inform future research and, if replicated, could help tailor support.

### 4.1 Spiritual well-being as a central connector

Our analysis shows that SpWB is not merely one among many factors associated with QoL; it is the most pivotal connector in terms of bridging physical, emotional, social, and functional life domains (34). This structural role shows SpWB as a central connector. Interventions or community programs designed to enhance spiritual well-being—particularly its meaning and peace dimensions—may be associated with positive changes across multiple aspects of a survivor’s life simultaneously (though causal direction cannot be inferred from cross-sectional data). This association suggests that integrating spiritual health support into survivorship care programs could be a strategy worth testing in future longitudinal or intervention studies to determine whether it yields broad-based well-being improvements (35). The strong connections observed between SpWB and nodes like “pride in disease resilience” and “preserved feminine identity” further indicate its importance in mitigating profound identity threats posed by cancer, a concern that psychosocial support services must address (36). The strong connection between SpWB and “preserved feminine identity” (AC_9) resonates with qualitative findings that breast cancer profoundly affects women’s sense of femininity and sexual self-concept (18). For younger survivors in particular, maintaining a positive body image and sexual identity is closely tied to overall well-being, suggesting that interventions aimed at enhancing spiritual well-being may also need to address body image and sexual health concerns.

The network was sparse (mean edge weight = 0.023, 34% of edges present). This sparsity does not indicate that QoL and SpWB domains are independent; rather, it suggests that their interconnections are not diffuse but are concentrated through a limited set of high-impact hub nodes—such as spiritual well-being, fear of disease progression, and disease acceptance. This “sparse but concentrated” topology is clinically meaningful: it implies that interventions targeting these specific associated hubs may achieve broad systemic effects without requiring simultaneous action on all fronts. From a public health perspective, this architecture supports the feasibility of precision support strategies, where limited resources can be directed toward nodes with the greatest potential for cross-domain impact.

### 4.2 Age differences in network connectivity

This study shows different well-being patterns between younger and older survivors. It is important to clarify that our network comparison tests showed no significant difference in the global network structure (the pattern of which nodes are connected; Network Invariance Test: *M* = 0.205, *p* = 0.831). This means that the basic “blueprint” of how quality of life and spiritual well-being domains associate with one another is similar across both age groups.

However, a significant difference emerged when we examined the intensity of cross-domain connectivity, quantified by node-level bridge strength. This metric measures the degree to which a node connects different life dimensions (e.g., linking emotional status to functional status). We found that bridge strength was substantially higher in younger survivors (*t* = 9.30, *p* < 0.001, Cohen’s *d* = 1.656). This indicates that while the pattern of connections is the same, the strength of those connections—particularly those bridging different domains—is greater in younger individuals. In other words, the well-being system of younger survivors is more “tightly interwoven” or “coupled,” making them more vulnerable to cascading effects where distress in one area quickly spreads to others. This nuance is essential for designing targeted interventions.

For younger survivors, the heightened bridge strength indicates that quality of life factors were more densely interconnected, with challenges related to identity, emotional distress, and family roles forming a central, tightly linked cluster (37). This pattern suggests that difficulties in one area (e.g., body image) may quickly amplify problems in others (e.g., family functioning), indicating a higher vulnerability to interconnected distress and a need for integrated, holistic support packages. This “tightly interwoven” architecture in younger survivors may be explained by the unique developmental tasks and social role expectations characteristic of this life stage. Individuals in their reproductive and career-building years often face the challenge of establishing intimate relationships, raising young children, and solidifying a professional identity (37). A breast cancer diagnosis can fundamentally disrupt the pursuit of these milestones, creating a cascading effect where threats to one domain—such as body image and femininity (AC_9)—directly and profoundly impact others, including emotional stability (ES_1) and role functioning within the family and workplace. Their well-being system is more “coupled” because their lives are more “coupled” with multiple, concurrently active social and developmental demands.

In contrast, the network for older survivors was sparser, with well-being more reliant on specific, discrete resources such as social and partner support. This pattern likely reflects the different psychosocial context of later life. For older survivors, the core developmental tasks often shift from achievement and identity formation to legacy, meaning-making, and adapting to age-related changes (38). While the threat of cancer is significant, it may not collide as forcefully with the central life tasks of this stage. Instead, their well-being may be more dependent on the continuity of established social roles and the strength of long-standing support systems. Furthermore, the higher likelihood of age-related comorbidities may mean that their overall health perception is partitioned into more distinct domains (e.g., “cancer-related issues” vs. “other health issues”), contributing to the sparser network structure. This points to different pathways to maintaining QoL and implies that support services for this group might be effectively focused on strengthening and leveraging these existing social networks, particularly as our findings and prior research underscore the pivotal role of partner and family dynamics for this group.

Our observation that older survivors’ well-being is more reliant on discrete social support nodes, such as family support and communication, is consistent with the findings of Maleki et al. (17), who emphasized the importance of partner support and family dynamics in the adjustment of breast cancer survivors and their husbands. These insights reinforce the need for couple-and family-based interventions, particularly for older couples where traditional support networks may play a pivotal role. This points to different pathways to maintaining QoL and implies that support services for this group might be effectively focused on strengthening and leveraging existing social networks. These differences suggest that uniform survivorship support models may be less efficient in this context, and provide preliminary evidence for considering age-stratified approaches in future research and program development within similar healthcare settings. For younger survivors, integrated psycho-spiritual interventions addressing identity, fear, and emotional regulation may be most effective. For older survivors, programs that strengthen existing social networks—particularly family and partner communication—may yield greater benefits. Tailoring resources in this way can enhance engagement and the effective allocation of community and clinical resources (38, 39).

### 4.3 Key nodes for future research

Network analysis identified fear of disease progression as the most central concern. The symptom “fear of disease progression” emerged as the most central and influential concern within the network. This suggests it may be a high-priority target for future community-based psychoeducation, screening, and intervention research. If future longitudinal studies confirm its role, addressing this fear might benefit mental health and overall adjustment. This finding aligns with prior qualitative research by Mardadni et al. (7), which identified fear of cancer recurrence as a dominant concern among breast cancer survivors and detailed the diverse coping strategies they employ, such as seeking social support and building resilience. The centrality of fear in our network underscores the need for interventions that not only address the fear itself but also leverage survivors’ existing coping resources, as recommended by Mardadni et al. Conversely, positive states like “disease acceptance” and “current life enjoyment” were also highly central, identifying them as critical assets to be recognized, validated, and fortified through supportive care programs and peer support networks. This dual focus—mitigating key distress points and bolstering key resilience points—offers a balanced strategy for public health action (40, 41).

### 4.4 Implications for future research

This study provides a novel, system-level perspective within a Chinese tertiary hospital context. To guide future research (rather than immediate practice changes), we propose the following considerations for investigation:

1 Consider integrating spiritual health assessment into future survivorship care research: routine, brief assessment of spiritual well-being (using tools like the FACIT-Sp-12) can help identify survivors for whom this is a key resource or area of need, facilitating connection to appropriate spiritual care or community resources.
2 Develop and disseminate age-tailored support resources: public health agencies and survivor support organizations should create distinct resource kits, workshop materials, and digital content that address the specific priority concerns of younger versus older survivor populations, as identified in this study.
3 Prioritize fear of progression in future research and program development: given its central role in the network, fear of disease progression should be a focus of future prospective studies and, if causal effects are supported, could be incorporated into support program design.
4 Future public health research should test the effectiveness of interventions designed from this network blueprint and explore how socioeconomic, cultural, and healthcare system factors further shape these well-being networks to ensure equitable support for all survivors.
5 In conclusion, by mapping the interconnected architecture of quality of life and spiritual well-being, this study moves the field from knowing that spirituality is important to understanding how it connects to broader well-being and for whom these connections matter most. These findings offer a pragmatic, evidence-based framework for developing more precise, efficient, and person-centered public health strategies to support the diverse and growing community of breast cancer survivors.

## 5 Limitations

The findings of this study should be interpreted within the context of its boundaries, which also delineate clear avenues for future public health research.

1 Causal direction and temporal dynamics. The cross-sectional design provides a detailed snapshot of the associations between QoL and SpWB at a specific point during active treatment. While this allows for the identification of hypothesized high-candidate targets within the system, it cannot establish causal direction or capture how these interrelationships evolve across the survivorship trajectory. Understanding temporal dynamics is crucial for determining the optimal timing of support interventions and for evaluating their long-term effectiveness in public health programming.
2 Generalizability and socio-cultural context. Our network was estimated from a cohort of Chinese breast cancer patients treated at a single tertiary center. This homogeneous sample facilitated a clear initial mapping of the architecture but necessitates validation in more diverse populations. The generalizability of this specific network structure to survivors in different cultural and spiritual frameworks, healthcare systems, and socioeconomic contexts remains to be tested. Such research is essential for developing equitable and context-sensitive public health models.
3 Measurement and construct aggregation of spiritual well-being. Spiritual well-being (SpWB) was modeled as a single aggregate node using the total score of the FACIT-Sp-12 scale. This decision was intentional and guided by two considerations. First, our primary aim was to examine whether global SpWB functions as a cross-domain bridge linking physical, emotional, social, and functional quality of life (QoL) domains—a macro-level question that does not require distinguishing among SpWB subdimensions. Second, maintaining a parsimonious network with 37 nodes (instead of 39 nodes if subdimensions were entered separately) enhanced estimation stability, as confirmed by our correlation stability coefficient (CS-coefficient = 0.75). However, we acknowledge that this aggregate approach may obscure potentially distinct roles of the three SpWB subdimensions—meaning, peace, and faith—in connecting to specific QoL domains. For example, the “peace” dimension might more strongly relate to emotional well-being, while “meaning” could be more tightly linked to functional status. Future research with larger samples (e.g., *N* > 500) should disaggregate SpWB to map the differential bridge functions of its subdimensions, which could inform more targeted spiritual care interventions. Additionally, cross-cultural validation of the FACIT-Sp-12 items in Chinese populations remains warranted, as expressions of spirituality may vary across cultural and religious contexts.
4 Finally, our sampling strategy focused exclusively on patients undergoing chemotherapy, which may limit the generalizability of findings to breast cancer survivors who receive other treatment modalities (e.g., endocrine therapy alone) or who are not receiving active treatment. Future research should include patients across the full spectrum of treatment trajectories to enhance the representativeness of the well-being network.

### 5.1 Future research directions

Building on these findings and limitations, we propose the following research agenda directly aimed at informing public health practice and policy:

1 Validation and comparison across diverse populations and systems. Replicating this network analysis in multi-center, international, and socioeconomically diverse cohorts of breast cancer survivors, as well as other cancer populations, is a critical next step. This work will help distinguish universal intervention targets from culture- or context-specific pathways, forming the foundation for equitable and inclusive public health support resources.
2 Design and evaluation of network-informed community interventions. Future research should develop and rigorously evaluate the effectiveness of community-based support programs explicitly designed to strengthen the key connector and central nodes identified here (e.g., workshops enhancing meaning/peace, CBT-based modules for fear of progression). Evaluation should include not only traditional scale scores but also metrics relevant to public health impact, such as changes in participants’ perceived interconnectedness of challenges or enhanced utilization of integrative coping resources.
3 Longitudinal network mapping in real-world community settings. Implementing repeated network assessments within community-based survivorship cohorts, from diagnosis through long-term survival, can track the natural evolution of the well-being architecture. This approach can identify critical transition periods (e.g., end of active treatment, return to work) where the network may be most malleable, providing empirical evidence for timing the rollout of public health support services.
4 Integrated mixed-methods research. Combining quantitative network analysis with in-depth qualitative interviews can explore how survivors personally experience the “connection mechanisms” identified (e.g., how spiritual peace tangibly influences family roles or the management of fear). This integration will provide rich context to the quantitative findings, ensuring that the design of support services is deeply aligned with the lived experiences of the population they intend to serve.

### 5.2 Implications

The findings of this study offer multi-layered implications for enhancing the public health response to breast cancer survivorship.

#### 5.2.1 For future research and program development (preliminary evidence)

- Consider testing stratified strategies in future implementation research: based on our findings, age-stratified approaches (integrated psycho-spiritual interventions for younger survivors; social network strengthening for older survivors) appear promising and warrant further evaluation in prospective, multi-center studies before clinical adoption.
- Explore the feasibility of integrating spiritual well-being assessment in research settings: brief spiritual health measures (e.g., FACIT-Sp-12) could be used in future observational or interventional studies to examine whether addressing spiritual needs improves other domains. Large-scale population implementation would require replication in diverse cohorts.

#### 5.2.2 For the research agenda

- Establish “System Resilience” as a core outcome in intervention science: when evaluating community-based support programs, researchers should consider including metrics that reflect changes in the underlying network architecture (e.g., reduced centrality of fear, increased bridge strength of social support) as complementary outcomes to traditional symptom scores. This provides direct evidence of strengthening the individual’s overall well-being system.
- Conduct comparative effectiveness research to optimize resource allocation: public health research should compare the real-world impact, cost-effectiveness, and reach of interventions targeting different network associated hubs (e.g., spiritual well-being vs. fear management). This evidence is crucial for policymakers and health administrators to make informed decisions about which programs to scale for population-level benefit.

#### 5.2.3 For policy and professional education

- Incorporate “Psycho-Oncology” and “Network Thinking” into Public Health Training: Curricula for community health workers, public health professionals, and oncology care providers should include training on the multidimensional needs of cancer survivors and the interconnected nature of well-being. This fosters a systemic perspective essential for designing and managing complex public health support programs.
- Advocate for the integration of evidence-based psychosocial support into public health guidelines and funding models: using evidence such as that provided here, advocates and professional organizations should work to include structured psychosocial and spiritual support interventions within national cancer control plans, public health guidelines, and health insurance reimbursement schemes. This systemic integration is key to ensuring sustainable and equitable access to these vital components of comprehensive survivorship care.

## 6 Conclusion

This study used network analysis to map connections between SpWB and QoL. SpWB was a central link among physical, emotional, social, and functional domains. Age differences appeared: younger survivors had tighter interconnections among identity, emotional distress, and other domains, suggesting they may need integrated support. Older survivors relied more on separate social support nodes. Fear of disease progression was the most central concern.

This research provides preliminary, hypothesis-generating evidence from a single-center Chinese cohort. Pending replication, the findings suggest that integrating spiritual health and age-tailored support may be promising research directions.

## Data Availability

The raw data supporting the conclusions of this article will be made available by the authors, without undue reservation.

## Supplementary material

SUPPLEMENTARY FIGURE 1 Comparison of network structure and influence by age level. **(A)** Network for 50 years old or below (*N* = 184), **(B)** Network for 50 years old or higher (*N* = 118), **(C)** Strength and Expected Influence by age level.

SUPPLEMENTARY FIGURE 2 Comparison of bridge strength by age level.

SUPPLEMENTARY FIGURE 3 Bootstrapped difference test of edge weights. The x-axis and y-axis represent individual edges within the quality of life and spiritual health network. Gray boxes indicate non-significant differences, while black boxes indicate significant differences.

SUPPLEMENTARY FIGURE 4 Bootstrapped difference test of the node strength centrality. The x-axis and y-axis represent individual nodes within the quality of life and spiritual health network. Strength centrality values are plotted on the diagonal. Gray boxes indicate non-significant differences, while black boxes indicate significant differences.

SUPPLEMENTARY FIGURE 5 Bootstrapped difference test of the node bridge strength centrality. These supplementary materials are essential for providing robust empirical support for the network analysis findings presented in the main manuscript. They offer granular statistical validation, such as bootstrapped stability tests (e.g., CS-coefficient = 0.75) and edge-weight accuracy analyses, which underpin the reliability of key conclusions—including the pivotal role of spiritual well-being as a bridge node (strength = 3.601) and age-specific vulnerabilities. Additionally, they enable deeper interrogation of the network topology, facilitating replication and extension of the study’s mechanistic insights into quality-of-life and spiritual-health interactions.
