# Supplementary figures and images for: Unveiling age-differentiated pathways: spiritual well-being links to quality of life in breast cancer survivors through network analysis
Source: Front Public Health. 2026 Jun 12;14:1782688. doi: 10.3389/fpubh.2026.1782688 (PMC13303212; doi:10.3389/fpubh.2026.1782688)

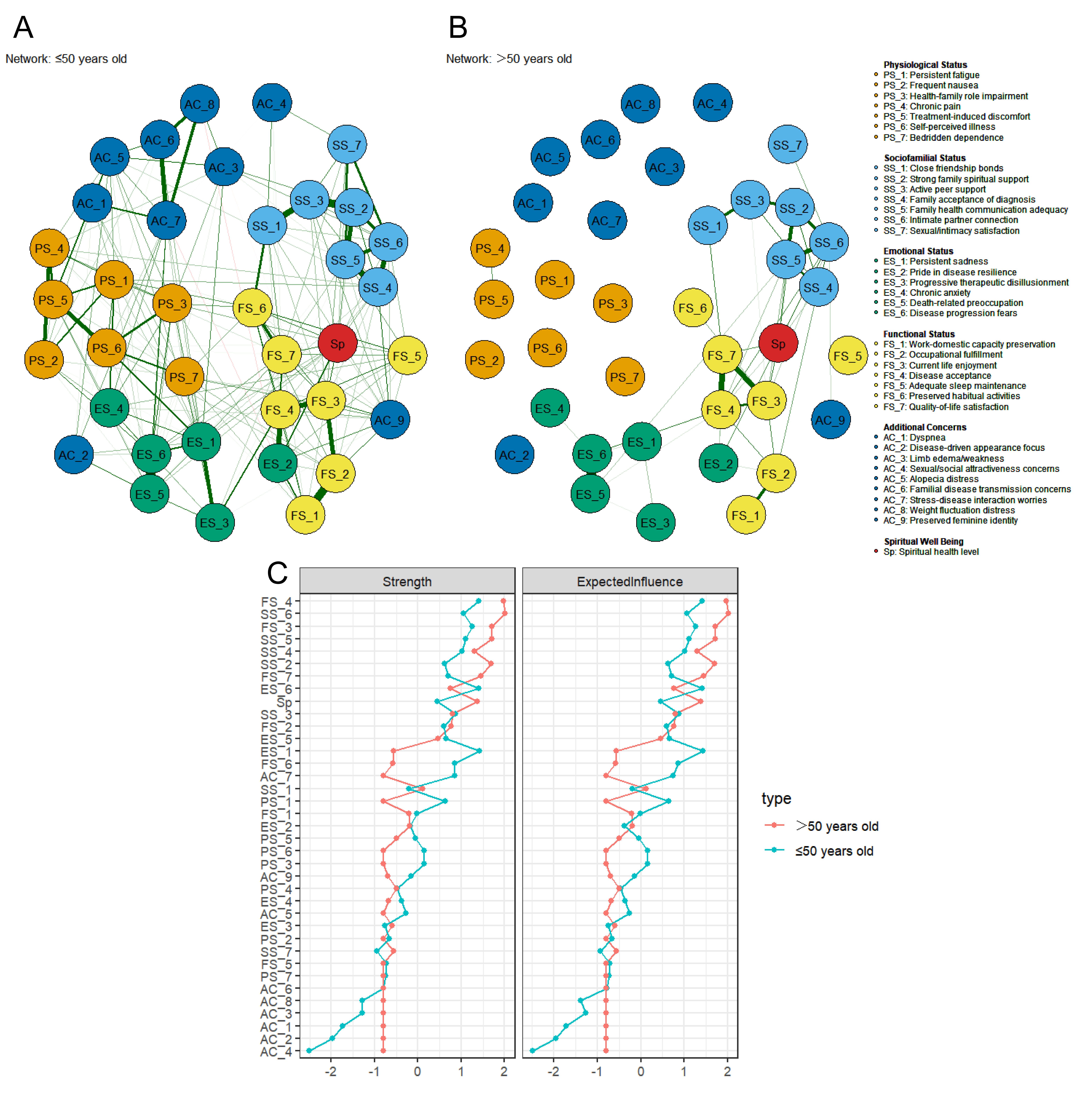

Supplement: SUPPLEMENTARY FIGURE 1 — Comparison of network structure and influence by age level. (A) Network for 50 years old or below (N = 184), (B) Network for 50 years old or higher (N = 118), (C) Strength and Expected Influence by age level. [file Image_1.png]

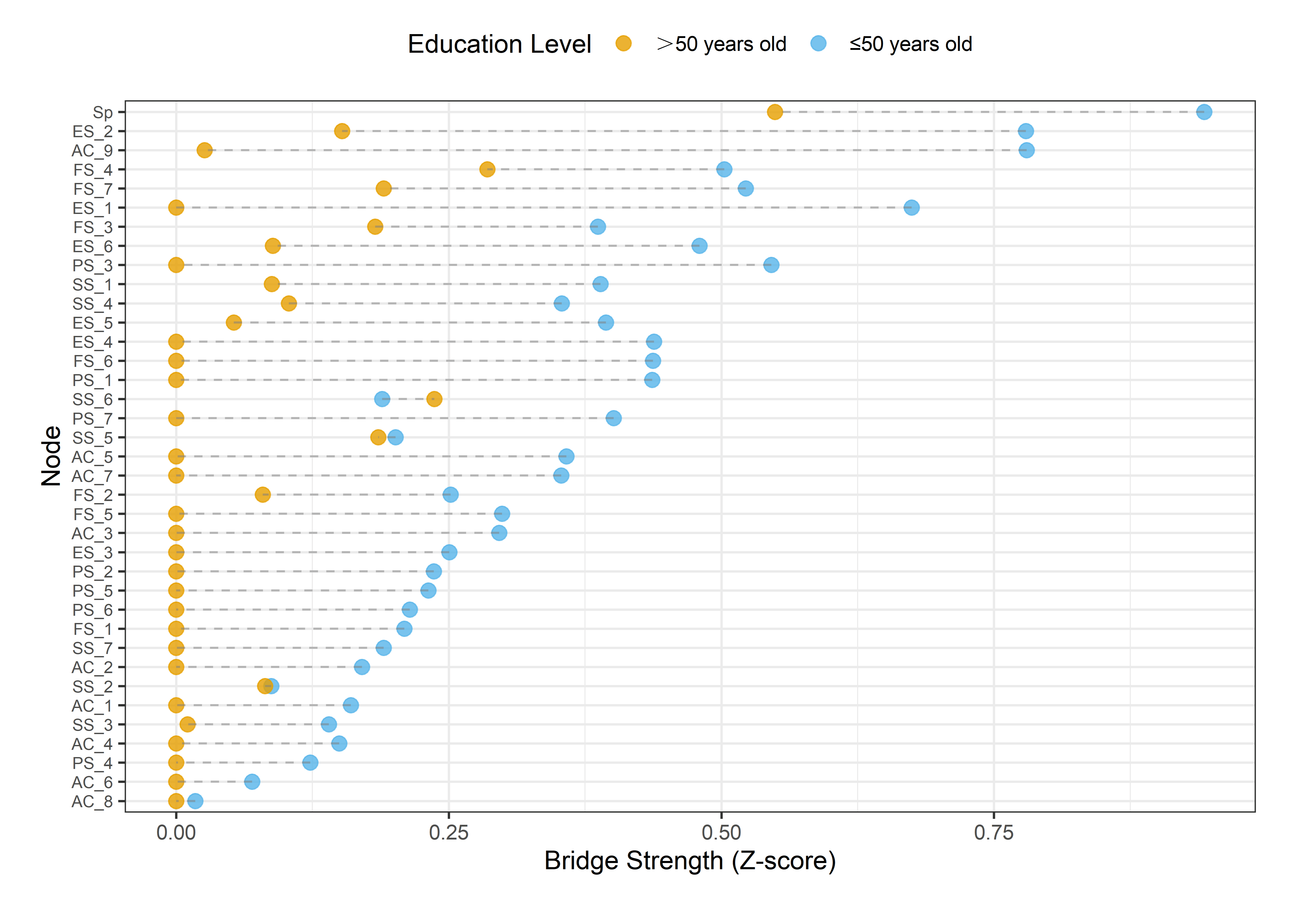

Supplement: SUPPLEMENTARY FIGURE 2 — Comparison of bridge strength by age level. [file Image_2.png]

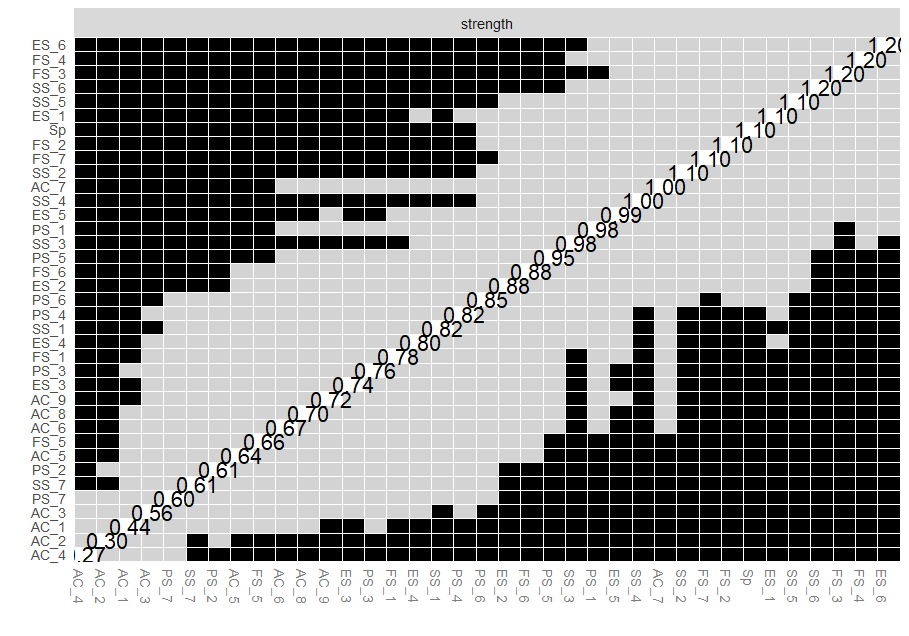

Supplement: SUPPLEMENTARY FIGURE 4 — Bootstrapped difference test of the node strength centrality. The x-axis and y-axis represent individual nodes within the quality of life and spiritual health network. Strength centrality values are plotted on the diagonal. Gray boxes indicate non-significant differences, while black boxes indicate significant differences. [file Image_4.tiff]

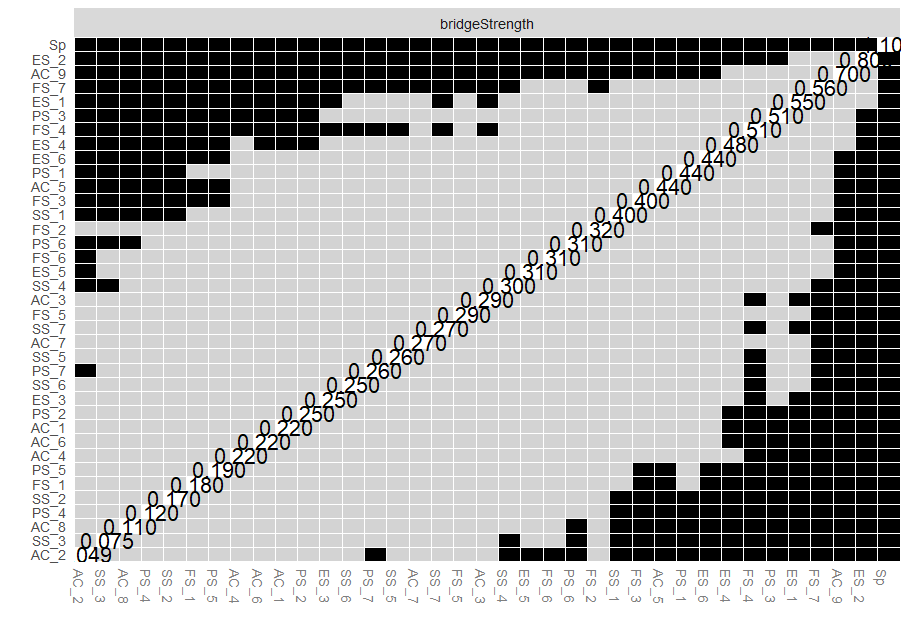

Supplement: SUPPLEMENTARY FIGURE 5 — Bootstrapped difference test of the node bridge strength centrality. These supplementary materials are essential for providing robust empirical support for the network analysis findings presented in the main manuscript. They offer granular statistical validation, such as bootstrapped stability tests (e.g., CS-coefficient = 0.75) and edge-weight accuracy analyses, which underpin the reliability of key conclusions—including the pivotal role of spiritual well-being as a bridge node (strength = 3.601) and age-specific vulnerabilities. Additionally, they enable deeper interrogation of the network topology, facilitating replication and extension of the study’s mechanistic insights into quality-of-life and spiritual-health interactions. [file Image_5.tiff]
