# Supplementary material for: Unveiling age-differentiated pathways: spiritual well-being links to quality of life in breast cancer survivors through network analysis
Source: Front Public Health. 2026 Jun 12;14:1782688. doi: 10.3389/fpubh.2026.1782688 (PMC13303212; doi:10.3389/fpubh.2026.1782688)
Supplement: Supplementary file 6 [file Table_1.docx]

Supplementary Table 1. **Comparison of general characteristics between the two age groups**

| **Characteristics** | **≤50 years（*N*=184)** | | **＞50 years（*N*=118)** | | Total | χ² | *P* value |
| --- | --- | --- | --- | --- | --- | --- | --- |
|  | N | % | N | % |  |  |  |
| **Ethnicity** |  |  |  |  |  |  |  |
| Han | 171 | 92.9% | 115 | 97.5% | 286 |  |  |
| Others | 13 | 7.1% | 3 | 2.5% | 16 | 2.931 | 0.087 |
| **Marital status*** |  |  |  |  |  |  |  |
| Married | 171 | 92.9% | 13 | 11.0% | 184 |  |  |
| Single | 110 | 59.8% | 8 | 6.8% | 118 | 0.009 | 0.924 |
| **Educational level** |  |  |  |  |  |  |  |
| Bachelor's degree or above | 72 | 39.1% | 16 | 13.6% | 88 |  |  |
| Junior college | 30 | 16.3% | 22 | 18.6% | 52 |  |  |
| Middle school and below | 82 | 44.6% | 80 | 67.8% | 162 | 23.595 | 0 |
| **Religious beliefs**** |  |  |  |  |  |  |  |
| Yes | 171 | 92.9% | 107 | 90.7% | 278 |  |  |
| No | 13 | 7.1% | 11 | 9.3% | 24 | 0.501 | 0.479 |
| **Current work status** |  |  |  |  |  |  |  |
| Retired | 30 | 16.3% | 76 | 64.4% | 106 |  |  |
| Employed | 93 | 50.5% | 12 | 10.2% | 105 |  |  |
| Unemployed | 61 | 33.2% | 30 | 25.4% | 91 | 82.526 | ＜0.001 |
| **Family residence location** |  |  |  |  |  |  |  |
| Urban | 129 | 70.1% | 84 | 71.2% | 213 |  |  |
| Rural | 35 | 19.0% | 16 | 13.6% | 51 |  |  |
| Town | 20 | 10.9% | 18 | 15.3% | 38 | 2.381 | 0.304 |
| **Residential status** |  |  |  |  |  |  |  |
| Living with family or friends | 180 | 97.8% | 4 | 3.4% | 184 |  |  |
| Living alone | 111 | 60.3% | 7 | 5.9% | 118 | 2.893 | 0.089 |
| **Level of family care and support since the illness onset** | | | | | | | |
| Very much | 139 | 75.5% | 95 | 80.5% | 234 |  |  |
| Quite a lot | 36 | 19.6% | 20 | 16.9% | 56 |  |  |
| Relatively little | 9 | 4.9% | 3 | 2.5% | 12 | 1.492 | 0.474 |
| **Personality types** |  |  |  |  |  |  |  |
| Ambivert type | 81 | 44.0% | 53 | 44.9% | 134 |  |  |
| Extroversion tendency type | 51 | 27.7% | 33 | 28.0% | 84 |  |  |
| Introversion tendency type | 28 | 15.2% | 13 | 11.0% | 41 |  |  |
| Typical extrovert type | 19 | 10.3% | 17 | 14.4% | 36 |  |  |
| Typical introvert type | 5 | 2.7% | 2 | 1.7% | 7 | 2.277 | 0.685 |
| **Per capita monthly household income** | | | | | | | |
| 5000 yuan and below | 72 | 39.1% | 45 | 38.1% | 117 |  |  |
| 5001-8000 yuan | 41 | 22.3% | 32 | 27.1% | 73 |  |  |
| 8001 yuan and above | 71 | 38.6% | 41 | 34.7% | 112 | 1.000 | 0.607 |
| **Disease duration (years)** |  |  |  |  |  |  |  |
| ＜1 | 168 | 91.3% | 108 | 91.5% | 276 |  |  |
| ≥1 | 16 | 8.7% | 10 | 8.5% | 26 | 0.004 | 0.947 |
| **Tumor stage** |  |  |  |  |  |  |  |
| Stage I | 68 | 37.0% | 46 | 39.0% | 114 |  |  |
| Stage II | 94 | 51.1% | 50 | 42.4% | 144 |  |  |
| Stage III | 22 | 12.0% | 22 | 18.6% | 44 | 3.43 | 0.18 |
| **Sequence of surgery and chemotherapy** |  |  |  |  |  |  |  |
| Primary debulking surgery (PDS) followed by chemotherapy | 157 | 85.3% | 106 | 89.8% | 263 |  |  |
| Neoadjuvant chemotherapy (NAC) followed by surgery | 27 | 14.7% | 12 | 10.2% | 39 | 1.297 | 0.255 |
| **Breast surgery approaches** |  |  |  |  |  |  |  |
| Mastectomy | 68 | 37.0% | 81 | 68.6% | 149 |  |  |
| Breast-conserving surgery | 70 | 38.0% | 32 | 27.1% | 102 |  |  |
| Breast reconstruction | 46 | 25.0% | 5 | 4.2% | 51 | 35.525 | ＜0.001 |
| **The total number of chemotherapy cycles required to be received** | | | | | | | |
| 8 | 60 | 32.6% | 31 | 26.3% | 91 |  |  |
| 6 | 49 | 26.6% | 34 | 28.8% | 83 |  |  |
| 4 | 74 | 40.2% | 52 | 44.1% | 126 |  |  |
| Other | 1 | 0.2% | 1 | 0.8% | 2 | 1.439 | 0.696 |
| **Chemotherapy completion status** | | | | | | | |
| Completed | 60 | 32.6% | 35 | 29.7% | 95 |  |  |
| Uncompleted | 124 | 67.4% | 83 | 70.3% | 207 | 2.900 | 0.590 |
| Note. *Single indicated separated, divorced, widowed, or never married, and married indicated married or partnered.  **Religious affiliations included Buddhism (22 individuals) and Taoism (2 individuals). | | | | | | | |
