# Supplementary material for: Unveiling age-differentiated pathways: spiritual well-being links to quality of life in breast cancer survivors through network analysis
Source: Front Public Health. 2026 Jun 12;14:1782688. doi: 10.3389/fpubh.2026.1782688 (PMC13303212; doi:10.3389/fpubh.2026.1782688)
Supplement: Supplementary file 7 [file Table_2.docx]

Supplementary Table 2. Functional Assessment of Cancer Therapy-Breast（FACT-B）and descriptive statistics for each question within the scale.

| **Items** | **Mean** | **SD** |
| --- | --- | --- |
| **Physiological Status(PS)** |  |  |
| PS_1 I experience fatigue/lack of energy | 2.77±0.87 | 2.77±0.87 |
| PS_2  I experience nausea | 3.20±0.83 | 3.20±0.83 |
| PS_3 Due to my poor physical condition, I am unable to meet my family's needs | 2.95±0.92 | 2.95±0.92 |
| PS_4 I experience pain | 2.81±0.89 | 2.81±0.89 |
| PS_5 The side effects of treatment cause me discomfort | 2.42±0.90 | 2.42±0.90 |
| PS_6 I feel ill | 2.62±0.95 | 2.62±0.95 |
| PS_7 I am confined to bed | 3.46±0.76 | 3.46±0.76 |
| **Sociofamilial Status(SS)** |  |  |
| SS_1 I maintain close relationships with my friends | 2.66±0.96 | 2.66±0.96 |
| SS_2 I receive emotional support from my family members | 3.21±0.79 | 3.21±0.79 |
| SS_3 I am supported by my friends | 2.95±0.85 | 2.95±0.85 |
| SS_4 My family has come to terms with my illness | 3.06±0.87 | 3.06±0.87 |
| SS_5 I am satisfied with the communication regarding my health condition within my family | 2.95±0.91 | 2.95±0.91 |
| SS_6 I feel emotionally connected to my partner (or the person I consider most significant) | 3.00±0.95 | 3.00±0.95 |
| SS_7 I am satisfied with my sexual life/intimate relationships | 2.44±1.08 | 2.44±1.08 |
| **Emotional Status(ES)** |  |  |
| ES_1 I feel sorrowful | 3.09±0.88 | 3.09±0.88 |
| ES_2 I take pride in my ability to confront this illness | 2.27±1.22 | 2.27±1.22 |
| ES_3 Throughout the disease trajectory, I experience escalating disillusionment | 3.58±0.69 | 3.58±0.69 |
| ES_4 I perceive heightened tension | 3.04±0.76 | 3.04±0.76 |
| ES_5 I harbor concerns about potential mortality | 3.15±0.85 | 3.15±0.85 |
| ES_6 I am apprehensive about disease progression | 3.00±0.86 | 3.00±0.86 |
| **Functional Status(FS)** |  |  |
| FS_1 I am able to work (including household work) | 2.37±0.98 | 2.37±0.98 |
| FS_2 My work (including household work) gives me a sense of accomplishment | 2.30±1.09 | 2.30±1.09 |
| FS_3 I am currently enjoying life very much | 2.52±1.06 | 2.52±1.06 |
| FS_4 I am able to accept my illness | 2.52±0.99 | 2.52±0.99 |
| FS_5 I sleep well | 1.82±1.16 | 1.82±1.16 |
| FS_6 I still engage in activities I used to do regularly | 2.22±1.03 | 2.22±1.03 |
| FS_7 I am satisfied with my current quality of life | 2.33±1.02 | 2.33±1.02 |
| **Additional Concerns(AC)** |  |  |
| AC_1 I experience shortness of breath | 3.34±0.81 | 3.34±0.81 |
| AC_2  I am concerned about my appearance/dress due to my illness | 2.76±1.13 | 2.76±1.13 |
| AC_3  I have swelling or weakness in one or both arms | 3.19±0.81 | 3.19±0.81 |
| AC_4 I feel less attractive sexually/in social interactions with peers | 1.16±0.93 | 1.16±0.93 |
| AC_5  I am troubled by hair loss | 2.33±1.18 | 2.33±1.18 |
| AC_6 I worry that other family members might develop the same illness as me someday | 2.86±1.05 | 2.86±1.05 |
| AC_7 I am concerned about the impact of stress on my condition | 2.75±0.94 | 2.75±0.94 |
| AC_8 Changes in my weight distress me | 3.17±0.98 | 3.17±0.98 |
| AC_9 I still feel like a woman | 2.49±1.13 | 2.49±1.13 |
| *Note:* SD: standard deviations. PS: Physiological Status, SS: Sociofamilial Status, ES: Emotional Status, FS: Functional Status, AC: Additional Concerns. | | |
