# Supplementary material for: Unveiling age-differentiated pathways: spiritual well-being links to quality of life in breast cancer survivors through network analysis
Source: Front Public Health. 2026 Jun 12;14:1782688. doi: 10.3389/fpubh.2026.1782688 (PMC13303212; doi:10.3389/fpubh.2026.1782688)
Supplement: Supplementary file 8 [file Table_3.docx]

Supplementary Table 3. The network between quality of life and spiritual health.

| **Nodes** | **Variables** | **Strength** | **Bridgestrength** | **Expectedinfluence** | **Bridge expected influence (1-step)** | **Predictability (R2)** |
| --- | --- | --- | --- | --- | --- | --- |
| PS_1 | Persistent fatigue | 0.525 | 0.426 | 0.586 | 0.535 | 0.271 |
| PS_2 | Frequent nausea | -0.957 | -0.485 | -0.895 | -0.564 | 0.183 |
| PS_3 | Health-related family role impairment | -0.360 | 0.811 | -0.198 | 0.905 | 0.053 |
| PS_4 | Chronic pain | -0.107 | -1.076 | 0.026 | -0.911 | 0.230 |
| PS_5 | Treatment-induced discomfort | 0.401 | -0.781 | 0.406 | -0.718 | 0.385 |
| PS_6 | Self-perceived illness | -0.008 | -0.177 | 0.113 | -0.045 | 0.341 |
| PS_7 | Bedridden dependence | -1.003 | -0.405 | -0.768 | -0.265 | 0.153 |
| SS_1 | Close friendship bonds | -0.134 | 0.276 | 0.002 | 0.391 | 0.483 |
| SS_2 | Strong family spiritual support | 0.909 | -0.853 | 0.925 | -0.697 | 0.698 |
| SS_3 | Active peer support | 0.521 | -1.315 | 0.582 | -1.141 | 0.629 |
| SS_4 | Family acceptance of diagnosis | 0.728 | -0.241 | 0.766 | -0.107 | 0.594 |
| SS_5 | Family health communication adequacy | 1.024 | -0.398 | 1.027 | -0.258 | 0.667 |
| SS_6 | Intimate partner connection | 1.330 | -0.444 | 1.299 | -0.302 | 0.654 |
| SS_7 | Sexual/intimacy satisfaction | -0.964 | -0.387 | -0.734 | -0.247 | 0.378 |
| ES_1 | Persistent sadness | 0.971 | 0.980 | 0.980 | 1.068 | 0.264 |
| ES_2 | Pride in disease resilience | 0.109 | 2.209 | -0.441 | 1.386 | 0.398 |
| ES_3 | Progressive therapeutic disillusionment | -0.417 | -0.456 | -0.249 | -0.314 | 0.284 |
| ES_4 | Chronic anxiety | -0.215 | 0.656 | -0.070 | 0.757 | 0.287 |
| ES_5 | Death-related preoccupation | 0.574 | -0.189 | 0.501 | -0.225 | 0.526 |
| ES_6 | Disease progression fears | 1.522 | 0.440 | 1.469 | 0.549 | 0.599 |
| FS_1 | Work-domestic capacity preservation | -0.283 | -0.807 | -0.343 | -0.932 | 0.461 |
| FS_2 | Occupational fulfillment | 0.950 | -0.119 | 0.692 | -0.345 | 0.567 |
| FS_3 | Current life enjoyment | 1.432 | 0.280 | 1.308 | 0.288 | 0.664 |
| FS_4 | Disease acceptance | 1.492 | 0.787 | 1.442 | 0.882 | 0.557 |
| FS_5 | Adequate sleep maintenance | -0.768 | -0.290 | -0.799 | -0.469 | 0.281 |
| FS_6 | Preserved habitual activities | 0.110 | -0.189 | 0.218 | -0.057 | 0.411 |
| FS_7 | Quality-of-life satisfaction | 0.915 | 1.030 | 0.931 | 1.116 | 0.495 |
| AC_1 | Dyspnea | -1.628 | -0.589 | -1.534 | -0.722 | 0.000 |
| AC_2 | Disease-driven appearance focus | -2.208 | -1.436 | -2.478 | -1.484 | 0.000 |
| AC_3 | Limb edema/weakness | -1.168 | -0.264 | -0.914 | -0.129 | 0.000 |
| AC_4 | Sexual/social attractiveness concerns | -2.320 | -0.601 | -2.551 | -0.843 | 0.000 |
| AC_5 | Alopecia distress | -0.819 | 0.425 | -0.605 | 0.534 | 0.146 |
| AC_6 | Familial disease transmission concerns | -0.734 | -0.591 | -0.769 | -0.758 | 0.264 |
| AC_7 | Stress-disease interaction worries | 0.729 | -0.393 | 0.609 | -0.460 | 0.363 |
| AC_8 | Weight fluctuation distress | -0.600 | -1.124 | -1.022 | -1.760 | 0.233 |
| AC_9 | Preserved feminine identity | -0.522 | 1.691 | -0.494 | 1.752 | 0.211 |
| SP | Spiritual health level | 0.525 | 3.601 | 0.980 | 3.591 | 0.456 |
