# Supplementary material for: Unveiling age-differentiated pathways: spiritual well-being links to quality of life in breast cancer survivors through network analysis
Source: Front Public Health. 2026 Jun 12;14:1782688. doi: 10.3389/fpubh.2026.1782688 (PMC13303212; doi:10.3389/fpubh.2026.1782688)
Supplement: Supplementary file 9 [file Table_4.docx]

Supplementary Table 4. Comparative analysis of bridge strength differences across age groups among 37 nodes

| **Node** | | **≤50 years** | **＞50 years** | **Difference** |
| --- | --- | --- | --- | --- |
| AC_9 | Preserved feminine identity | 0.780 | 0.026 | 0.754 |
| ES_1 | Work-domestic capacity preservation | 0.675 | 0.000 | 0.675 |
| ES_2 | Occupational fulfillment | 0.779 | 0.152 | 0.627 |
| PS_3 | Health-related family role impairment | 0.546 | 0.000 | 0.546 |
| ES_4 | Chronic anxiety | 0.438 | 0.000 | 0.438 |
| FS_6 | Preserved habitual activities | 0.437 | 0.000 | 0.437 |
| PS_1 | Persistent fatigue | 0.437 | 0.000 | 0.437 |
| PS_7 | Bedridden dependence | 0.401 | 0.000 | 0.401 |
| SP | Spiritual health level | 0.943 | 0.549 | 0.394 |
| ES_6 | Disease progression fears | 0.480 | 0.089 | 0.391 |
| AC_5 | Alopecia distress | 0.358 | 0.000 | 0.358 |
| AC_7 | Stress-disease interaction worries | 0.353 | 0.000 | 0.353 |
| ES_5 | Death-related preoccupation | 0.394 | 0.053 | 0.341 |
| FS_7 | Quality-of-life satisfaction | 0.522 | 0.190 | 0.332 |
| SS_1 | Close friendship bonds | 0.389 | 0.088 | 0.302 |
| FS_5 | Adequate sleep maintenance | 0.299 | 0.000 | 0.299 |
| AC_3 | Limb edema/weakness | 0.296 | 0.000 | 0.296 |
| ES_3 | Progressive therapeutic disillusionment | 0.251 | 0.000 | 0.251 |
| SS_4 | Family acceptance of diagnosis | 0.354 | 0.103 | 0.250 |
| PS_2 | Frequent nausea | 0.236 | 0.000 | 0.236 |
| PS_5 | Treatment-induced discomfort | 0.231 | 0.000 | 0.231 |
| FS_4 | Disease acceptance | 0.503 | 0.285 | 0.217 |
| PS_6 | Self-perceived illness | 0.214 | 0.000 | 0.214 |
| FS_1 | Work-domestic capacity preservation | 0.209 | 0.000 | 0.209 |
| FS_3 | Current life enjoyment | 0.387 | 0.183 | 0.204 |
| SS_7 | Sexual/intimacy satisfaction | 0.190 | 0.000 | 0.190 |
| FS_2 | Occupational fulfillment | 0.252 | 0.079 | 0.172 |
| AC_2 | Disease-driven appearance focus | 0.170 | 0.000 | 0.170 |
| AC_1 | Dyspnea | 0.160 | 0.000 | 0.160 |
| AC_4 | Sexual/social attractiveness concerns | 0.150 | 0.000 | 0.150 |
| SS_3 | Active peer support | 0.140 | 0.011 | 0.130 |
| PS_4 | Chronic pain | 0.123 | 0.000 | 0.123 |
| AC_6 | Familial disease transmission concerns | 0.070 | 0.000 | 0.070 |
| SS_6 | Intimate partner connection | 0.189 | 0.237 | -0.048 |
| AC_8 | Weight fluctuation distress | 0.018 | 0.000 | 0.018 |
| SS_5 | Family health communication adequacy | 0.201 | 0.185 | 0.016 |
| SS_2 | Strong family spiritual support | 0.087 | 0.082 | 0.006 |
